# Hemorrhagic Gastric Ulcer due to the Stagnation of Rice Cake

**DOI:** 10.31662/jmaj.2018-0043

**Published:** 2019-02-01

**Authors:** Shogo Matsuda, Masaki Nishitani, Uichiro Fuchizaki

**Affiliations:** 1 Department of Gastroenterology, Keiju Medical Center, Nanao, Japan

**Keywords:** gastric foreign body, hemorrhagic gastric ulcer, rice cake

A 62-year-old man visited our hospital because of epigastric pain and black stools. He had a history of successful *Helicobacter pylori* eradication. Computed tomography (CT) revealed a 33-mm diameter mass in the stomach (Figure 1A). Esophagogastroduodenoscopy revealed a solid, whitish object (Figure 1B) and two gastric ulcers in the antrum (Figure 1C). One of the ulcers had a visible vessel (Figure 1D), and hemostatic clips were applied. The whitish object was thought to be hardened rice cake because of its high density on CT ^(1)^ and his history of swallowing a whole rice cake one month ago. We cut the rice cake into pieces with a snare and retrieved it. Thereafter, his symptoms rapidly resolved.

A freshly-cooked rice cake is soft and viscous. However, it solidifies at body temperature and may not pass through the pyloric ring if a large piece is swallowed. In the present case, the rice cake had stagnated for a month, and hemorrhagic gastric ulcers developed because of the mechanical stimuli ^(1)^.

**Figure 1.**
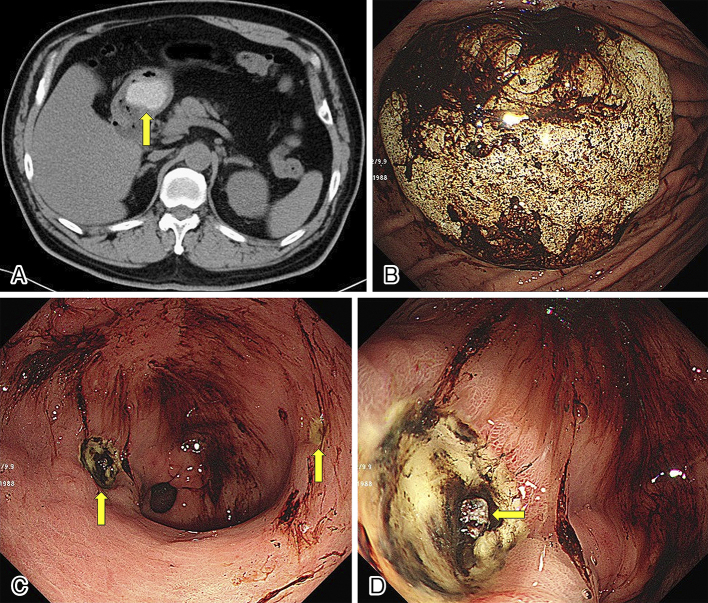
(A) Abdominal computed tomography scan showing a high-density mass (arrow) in the stomach. (B) Esophagogastroduodenoscopy (EGD) showing a whitish solid structure with hematin in the gastric body. (C) EGD showing two gastric ulcers (arrows) and hematin in the antrum. (D) One of the gastric ulcers had a visible vessel (arrow), and hemostatic clips were applied.

## Article Information

### Conflicts of Interest

None

### Author Contributions

S. Matsuda acquired data and drafted the manuscript. M. Nishitani and U. Fuchizaki reviewed and supervised the manuscript.

### Informed Consent

We obtained written consent of the patient.
